# Job stress and burnout among ideological and political education teachers during the COVID-19 pandemic: A moderated mediation model

**DOI:** 10.3389/fpsyg.2022.1008854

**Published:** 2022-10-18

**Authors:** Weiwei Shang

**Affiliations:** School of Marxism, East China Normal University, Shanghai, China

**Keywords:** job stress, job burnout, work–family conflict, resilience, ideological and political education teachers, pandemic

## Abstract

Job burnout among ideological and political education (IPE) teachers in China is a complex problem and rewarding area of research. This study explored the relationship between job stress and burnout among ideological and political education (IPE) teachers in the context of the COVID-19 pandemic. The cross-sectional design included a sample of self-report measures sampled from 362 university IPE teachers. Using multiple line regression analysis, our main findings were as follows: first, job stress had a significant predictive effect on work–family conflict and job burnout; second, work–family conflict mediated the relationship between job stress and job burnout; and finally, resilience productively moderated the relationships between job stress and both work–family conflict and burnout. These results suggest that resilient IPE teachers are less likely to suffer from burnout. They indicate the need to systematically foster resilience in trainees and experienced instructors as a means of coping with adverse work conditions.

## Introduction

Job stress and burnout impact a wide range of occupations (Schaufeli et al., 2009), and their prevalence among teachers, due to changes in the overall educational environment, is a source of global concern (Siegall and McDonald, 2004). Burnout syndrome is particularly widespread among members of the teaching profession (Padilla and Thompson, 2016; Fathi et al., 2021), and often worsens over time (McIntyre et al., 2017). The recent COVID-19 pandemic has exacerbated the issue (Dima et al., 2021; Alqassim et al., 2022): Chinese government measures to stop the spread of the virus have required teachers to adapt rapidly to remote teaching, raising levels of stress and increasing burnout rates (Besser et al., 2020; Gutentag and Asterhan, 2022).

In China, ideological and political education (IPE) teachers provide compulsory courses in the subject to the country’s university students. However, the quickening pace of reform in Chinese universities and increasing demands on academic staff are raising rates of burnout to levels that outstrip those of their colleagues in Western universities. The job of IPE teachers is to provide compulsory courses in ideological and political education to university students in China. For several reasons, IPE teachers experience higher levels of job stress: they receive lower professional recognition and heavier teaching loads including public required course at univerisity and professional course at school of Marxism, and the IPE curriculum is constantly updated, and students habitually undervalue the subject (Xiaodong and Bo, 2009). These difficulties have been compounded by the transition to online education during the pandemic, which has restricted the time available for rest and family activities. For many teachers, work–family balance has been lost due to the difficulty of reconciling the demands of each domain (Li et al., 2015). Faced with such conflicts and ambivalence, the personal resources of teachers may be depleted and they may suffer from increasing emotional exhaustion (Perry et al., 2010).

Measures to contain COVID-19 drastically changed lives of both students and teachers. School closures and at-home quarantines, the most widely used measures at the start of the pandemic (Esposito and Principi, 2020), affected 63 million teachers in 165 countries. Globally, 1.3 billion learners participated in alternative school experiences, such as virtual and hybrid learning models (Joshi et al., 2020). The mental health and wellbeing of teachers (and other professionals) declined due to the isolating and challenging nature of remote and working practices (Kaden, 2020; Marshall et al., 2020; Pressley, 2021), and teaching became more cognitively and emotionally taxing during the pandemic due to the challenges of teaching from home (Kim and Asbury, 2020). For example, teachers experienced inadequate support for the move from classroom teaching to online platforms; many were exposed to external distractions and family interruptions throughout the academic day and faced logistical barriers to conducting valid and reliable assessments (Joshi et al., 2020). In recent surveys, teachers have consistently reported medium-to-high levels of stress and burnout (Jakubowski and Sitko-Dominik, 2021; Alqassim et al., 2022).

The emotional resilience of teachers plays a pivotal role in how and whether they have experienced stress and burnout during the COVID-19 pandemic. However, the precise relationship between work–family conflict, work-based stress and burnout remains unclear, and the impact of family environment on teacher burnout is thus worthy of further investigation. This study aimed to bridge the current gap in knowledge by examining how job stress influences burnout among IPE teachers in the Chinese cultural context. This focus affords a more comprehensive and rational view of current working practices in education to alleviate or prevent teacher burnout and thus improve human resource management outcomes.

## Literature review and development of hypotheses

### Job stress and burnout

*Work stress* denotes any negative experience resulting from imbalances between the demands of a job and the resources available to workers (Hakanen et al., 2006). Stress caused by adverse working conditions damages individuals by reducing the quality of their work lives, lowering job satisfaction, eroding their motivation and ultimately causing burnout. However, it also impacts organizations by triggering increased sick leave, and additional compensation claims, lowering productivity and increasing rates of staff turnover (Hart and Cooper, 2001). Job stress occurs when the individual worker is unable to cope with the demands of their work (Schaufeli and Enzmann, 1998). Teacher job stress refers to negative experiences in the teaching environment (Kyriacou and Sutcliffe, 1977), which can lead to frustration with heavy workloads or excessive working hours, and then to nervous tension, physical and mental fatigue, and other negative health outcomes (Roeser et al., 2013). Evidence strongly suggests that teaching ranks among the most stressful jobs (Greenier et al., 2021), and such stress has been compounded by the pandemic (Ozamiz-Etxebarria et al., 2021).

*Job burnout* is a comprehensive manifestation of emotional exhaustion, depersonalization, and reduced personal accomplishment (Leiter et al., 2014). The Job Demands-Resources (JD-R) model indentifies two impairment conditions implicated in burnout: inadequacy of resources to cover work-related demands and exhaustion caused by heavy work loads (Demerouti et al., 2001). Teachers are particularly vulnerable to burnout (Zhong et al., 2009) due to imbalances between resources and demandsthat affect their psychological well-being at work (Prieto et al., 2008). Many studies showing that burnout is implicated in teachers’ stress responses (Siegall and McDonald, 2004) have utilized Maslach’s Burnout Scale, which consists of three measurement dimensions: emotional exhaustion, depersonalization and reduced fulfillment (Maslach, 1982). Examining the effects of job stress on burnout not only provides a better picture of how people work in a given occupational environment but also builds insight into the mechanisms of interaction between individuals and their external environments.

Previous studies have demonstrated that job stress and teacher burnout are positively correlated (Bottiani et al., 2019) with the latter more likely to occur when short-term stress is unalleviated (Lloyd et al., 2009). Unaddressed over the long term, negative work experiences often lead to burnout and poor mental health, in turn affecting the productivity of the whole organization. Chronic stress may lead to chronic fatigue, whose symptoms closely resemble those of burnout (Johnson et al., 2020). Individual levels of anxiety often risein response to pressures such as excessive work demands, loss of resources and disproportionately low returns on effort and resource allocation (Hobfoll, 1989). Among teachers, job stress and burnout are primary causes of attrition (Gallant and Riley, 2020) and reduce educational quality (Liu and Onwuegbuzie, 2012). Under the pressure of educational reform and the pandemic, the levels of stress reported by teachers have risen (Ozamiz-Etxebarria et al., 2021), with IPE teachers particularly affected owing to the perceived importance of their mission (Xiaodong and Bo, 2009). Based on these research findings, this study hypothesized that job stress would predict burnout among Chinese university IPE teachers.

*Hypothesis 1*: Job stress positively and significantly predicts job burnout.

### The mediating role of work–family conflict

Resource conservation theory (Hobfoll, 1989) is highly influential in the field of stress research. It describes how individuals manage their resources to cope with environmental stressors and how these influence their subsequent responses. The theory claims that people usually attempt to acquire, retain, and maintain their own resources, but when these are limited, individuals cannot fully meet workplace demands and the resulting psychological pressure can lead to burnout (Hobfoll, 2002). Work and family are two important components of social structure that should occupy balanced roles in people’s lives (Brosch and Binnewies, 2018). Owing to its importance in the Chinese cultural context, the study of work–family balance has received considerable academic and management attention. When educators are exposed to manifold challenges from teaching workloads, exams, student management, research duties and many other areas of their professional lives, they are likely to experience a sense of powerlessness or even frustration due to *work–family conflict* (WFC).

WFC refers to the internal role conflict arising when one role at home interferes with the performance of another at work, or vice versa(Greenhaus and Beutell, 1985). There are two types of WFC: interference with family due to work needs and interference with work due to family needs (Wu et al., 2009). As resource conservation theory highlights, resources are finite, so investing themin one role will limit their availability in another role; the relative or actual loss of resources is a major component of stress (Edwards and Rothbard, 2000). Individuals who lose work resources due to increased job stress or blurred work roles—whether subjectively perceived or actual—may also experience work–family conflict (Jin et al., 2013). Because the boundary between work and family contexts is permeable, problems encountered by teachers at work are likely to transfer to family life (Richter et al., 2015).Job stress and work–family conflict are linked (Ismail and Gali, 2017), with the former affecting how some individuals treat their spouses and children, exacerbating conflicts between work and family (Carlson et al., 2018).

In turn, such conflicts can further drain workers’ resources, leading to negative outputs. Many studies underline the links betweenwork–family conflict and job burnout, as individuals struggle to cope with the dual roles of work and family (Richter et al., 2015; Pu et al., 2017; Ji and Yue, 2020; Lambert et al., 2022). Family and work are important individual resources for IPE teachers and when they conflict, additional stress is generated, further impacting the balance between them (Lambert et al., 2022) and exacerbating the tendency toward burnout (Simães et al., 2021). On this basis, this study predicted that work–family conflict would mediate the relationship between job stress and burnout among IPE teachers.

*Hypothesis 2*: Work–family conflict mediates the relationship between job stress and burnout.

### The moderating role of resilience

Given the significant impact of burnout on teachers’ health, interventions have traditionally focused on reducing or eliminating factors that may contribute to the syndrome. However, this predominantly reactive approach has been progressively replaced by a more proactive perspective that focuses on the psychology of sustainability and sustainable development (Di Fabio, 2017a). From a theoretical standpoint, external attributions focus on the influence of objective environmental variables, while internal attributions belong to the internal subjective dimension, and the lack of a direct connection between the two is often manifested as a moderating mechanism between causal variables. That is, in the same environment, internal attributions such as an individual’s self-efficacy, personality endowment, psychological capital, etc. may moderate the relationship between stress and burnout, producing variable manifestations of burnout among group members. The importance of resilience in this regard has recently been recognized by psychology researchers (Beltman et al., 2011), with one study finding that resilient teachers tended to persevere under adverse conditions and adapted more easily to change (Mansfield et al., 2012). In other words, resilience may help individuals cope with stress, adjust their overall resource levels and influence work–family relationships, thus alleviating burnout and maintaining their sustained passion for teaching. However, the moderating effect of resilience on teacher burnout has rarely been studied.

The proliferation of research in the last 10 years on this topic indicates that resilience is a major form of psychological capital that positively contributes to mental health (Culbertson et al., 2010) and reduces the incidence of conditions such as burnout (Wang et al., 2012). Increased resilience makes individuals more adaptable to negative life events or emergencies contributing to positive functioning and preventing negative emotional states characteristic of burnout (Yıldırım and Solmaz, 2020). It also protects against the symptoms of stress, burnout and vicarious traumatisation (Back et al., 2016). Researchers concur that teacher resilience is a multi dimensional and dynamic construct. Precise definitions vary, but the term denotes the ability of educators to successfully manage challenges and maintain a commitment to educational practice through the interaction between personal attributes and the external environment (Brunetti, 2006). Based on the findings summarized above, this study postulated that resilience would mediate the linkage between job stress and burnout among IPE teachers.

*Hypothesis 3*: Resilience moderates the direct relationship between job stress and burnout among IPE teachers and the first half of the mediated path.

**Figure 1:**
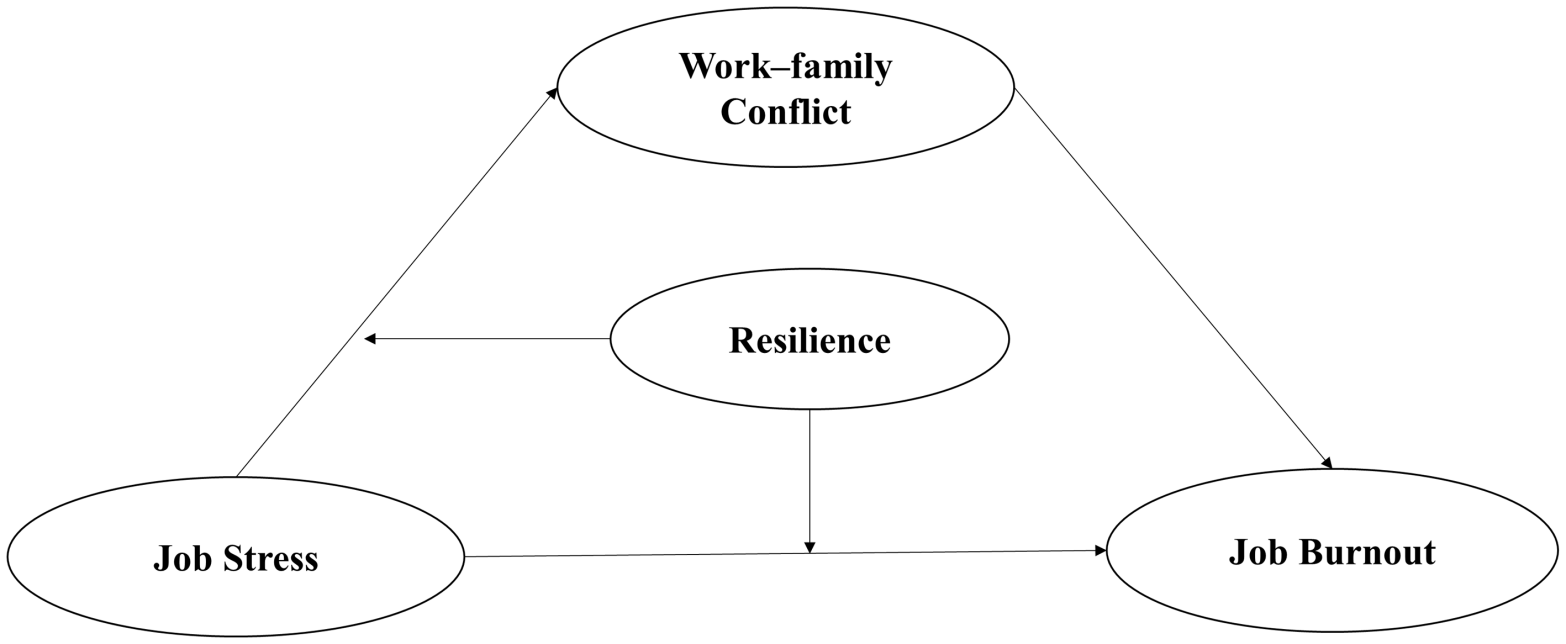
The Proposed moderated mediation model.

Our proposed conceptual model (Figure 1) was devised to explore the mediating and moderating factors influencing the relationship between job stress and burnout among IPE teachers and thereby support evidence-based interventions to make these and other educators more resistant to burnout.

## Materials and methods

### Participants and procedure

The data for this study were collected between April and May 2022 from university IPE teachers based in Shanghai. Because the city was under COVID-19 lockdown measures, all university teachers were isolated at home and teaching online. The study was approved by the ethics committee of the institution with which the study’s authors are affiliated. Data were gathered using an online survey hosted on the Wenjuanxing platform. The link shared with participants contained the questionnaire and instructions for completing it. Also included were guidelines informing teachers that their participation would be voluntary, anonymous and confidential. Participants could take as long as needed to complete the questionnaire on the web platform.

A random sample of IPE teachers from Shanghai universities returned 362 responses once 21 questionnaires containing missing values had been removed. All participants were aged 24–63 (*M* = 43.17; *SD* = 13.21; 257 women and 105 men); approximately 63% had a postgraduate qualification and 37% an undergraduate degree. In terms of experience, 12 participants (3.4%) had been teaching for less than 1 year, 155 (42.9%) between 1 and 5 years, 95 (26.2%) between 5 and 10 years, 60 (16.5%) between 10 and 20 years, 25 (6.9%) between 20 and 30 years, and 15 (4.1%) for more than 30 years.

### Measures

#### Teachers’ stressor scale

To gather data on teachers’ stress, this study used Oros et al.’s Scale of Teachers’ Stressors in Times of Pandemic (Oros et al., 2020), translated into Chinese by a Ph.D. student of English. This instrument has 21 items evaluating five components of teacher stress linked to pan-demic conditions: first, the working environment and work overload (sample item: “Having little time to do all the tasks involved in remote work”); second, the use of new technologies (e.g., “Learning how to use and master new technologies”); third, uncertainty about the duration and consequences of the pandemic for teachers and students (e.g., “Feeling uncertain toward the future, not knowing when the pandemic will end”); fourth, the organizational aspect of the educational institution (e.g., “Feel-ing that superiors do not understand the difficulties of working under these conditions”); and finally, the relationship with the student’s environment, conflict and role ambiguity (e.g., “Receiving multiple and simultaneous inquiries from students or/and parents”). Responses to the items were recorded on a five-point Likert-type scale ranging from 1 = Not stressful to 5 = Very stressful. The Cronbach’s alpha scores demonstrated sufficient internal consistency of between 0.78 and 0.85 for each factor and 0.93 for the full scale.

#### Teachers’ burnout questionnaire

To measure teacher burnout, this study used Wu et al.’s version (Wu et al., 2016) of the Maslach Burnout Inventory-Educators Survey (MBI-ES) (Maslach and Jackson, 2016), adapted to explore feelings associated with the Chinese context of the pandemic. This instrument consisted of 22 items, across three dimensions: emotional exhaustion (sample item as translated by a Ph.D. student of English: “I feel emotionally exhausted by my work”); depersonalization (“I have become more insensitive towards people since I started teaching”); and personal accomplishment (“I think I deal effectively with my students’ problems”). And, responses were given on a five-point Likert-type scale with scores ranging from 0 (never) to 6 (every day). The Cronbach’s alpha scores ranged from 0.72 to 0.90 for each factor and 0.91 for the full scale, demonstrating adequate levels of internal consistency.

#### Work–family conflict questionnaire

Wu et al.’s Chinese work–family conflict questionnaire was used to gather data on this issue during the pandemic (Wu et al., 2009). Adapted to the Chinese context and translated by a Ph.D. student of English, this instrument consisted of 22 items, divided into two dimensions: work interfering with family (“Things at work have put me in a bad mood and affected the family atmosphere”) and family interfering with work (“Conflicts with my family make me feel bad at work”). Responses’ feelings associated with pandemics. And, responses were recorded on a five-point Likert-type scale of 1 (rarely) to 5 (always). The two factors demonstrated adequate internal consistency with Cronbach’s alphas of 0.85 and 0.89, respectively. The internal consistency of the full scale was 0.91.

#### Resilience scale

To measure resilience, this study utilized Yu and Zhang’s Chinese adaptation (Yu and Zhang, 2007) of Connor and Davidson’s Maslach Burnout Inventory-Educators Survey (MBI-ES) (Connor and Davidson, 2003). Adapted to gauge our respondents’ feelings during the pandemic, this instrument consisted of 25 items, divided into three dimensions: Tenacity (“calmness and determination in the face of challenges”); Strength (“The ability to not only recover but also develop and grow through setbacks”); and Optimism (“Confidence in overcoming adversity, seeing things in a positive light”). Responses’ feelings associated with pandemics. And, responses were recorded on a five-point Likert-type scale ranging from 1 (rarely) to 5 (always). The Cronbach’s alpha scores of between 0.86 and 0.90 demonstrated the reliability of each factor, as did the scale’s overall internal consistency of 0.91.

### Data analysis

The analytic strategy proceeded in three stages. First, the main descriptive statistics and correlations among all variables were computed. Second, the PROCESS macro for SPSS was used to examine the mediating effect of work–family conflict. Third, the moderated mediating effect of resilience on the direct path and the first half of the mediating path was tested using the PROCESS macro for SPSS (Hayes, 2013). In addition, any gaps in the data were processed using maximum likelihood estimation.

## Results

### Common-method bias test

Because the data were derived from self-report instruments, it was important to check that common method bias was not present in the data. Accordingly, Harman’s single-factor test and the method-factor approach were used (Xing et al., 2012). The results showed that nine factors had eigenvalues of >1, with the first factor explaining 25.19% of the total variance, well below the recommended threshold of 40%. These investigations confirmed the absence of serious common method bias.

### Correlations between primary variables

The descriptive statistical results including Spearman’s correlations, means and SDs are presented in Table 1. As the table shows, job stress was positively correlated with work–family conflict (*r* = 0.492, *p* < 0.001) and job burnout (*r* = 0.584, *p* < 0.001). Moreover, work–family conflict was positively correlated with job burnout (*r* = 0.392, *p* < 0.001), whereas job stress was negatively correlated with resilience (*r* = −0.291, *p* < 0.001), and resilience was negatively correlated with work–family conflict (*r* = −0.305, *p* < 0.001) and job burnout (*r* = −0.316, *p* < 0.001).

**Table 1:** Descriptive statistics and correlation among variables.

Measures	*M*	*SD*	1	2	3	4
1. Job stress	3.21	1.028	1.000			
2. WFC	3.16	1.003	0.492^***^	1.000		
3. Resilience	3.42	1.209	−0.291^***^	−0.305^***^	1.000	
4. Job burnout	2.94	0.901	0.584^***^	0.392^***^	−0.316^***^	1.000

***p < 0.001; WFC, work–family conflict.

### Mediating effect analysis

This study investigated the predictive effect of teacher job stress on burnout, and the mediating role of work–family conflict using the PROCESS macro. Table 2 demonstrates that job stress was positively associated with job burnout (*β* = 0.49, *t* = 15.29, *p* < 0.001) in Model 1 and work–family conflict (*β* = 0.52, *t* = 16.14, *p* < 0.001) in Model 2. When the variable of work–family conflict was added to build Model 3, the positive direct association between job stress and burnout remained significant (*β* = 0.25, *t* = 4.35, *p* < 0.001), and work–family conflict was still positively related to job burnout (*β* = 0.46, *t* = 7.16, *p* < 0.001). Therefore, Hypotheses 1 and 2 were supported. Work–family conflict partially mediated the relationship between job stress and burnout (indirect effec*t* = 0.24, *SE* = 0.03, 95% CI = [0.21, 0.59]). Moreover, the mediation effect accounted for 49% of the total effect of job stress on burnout among IPE teachers.

**Table 2:** Testing the mediation effect of job stress on burnout.

Predictors	Model 1 (job burnout)		Model 2 (WFC)		Model 3 (job burnout)	
	*β*	*t*	*β*	*t*	*β*	*t*
Job stress	0.49^***^	15.29	0.52^***^	16.14	0.25^***^	4.35
WFC					0.46^***^	7.16
R2	0.18		0.41		0.26	
F	134.65^***^		384.02^***^		96.37^***^	

***p < 0.001; WFC, work–family conflict.

### Moderated mediation effect analysis

**Figure 2:**
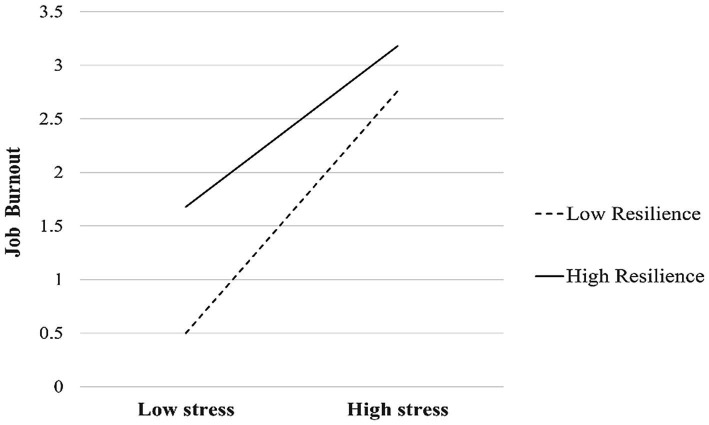
Resilience as a mediator between job stress and burnout.

**Figure 3:**
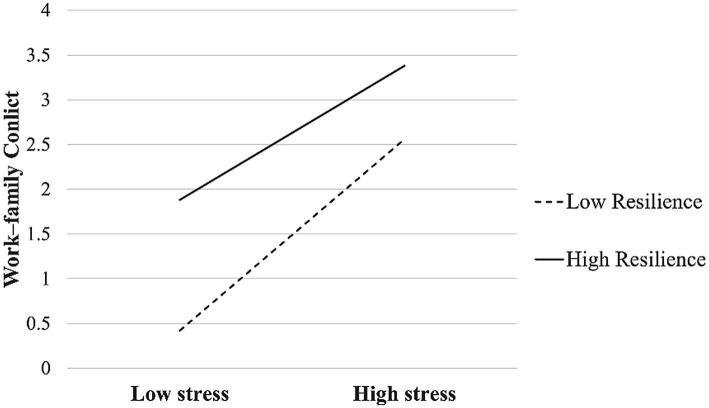
Resilience as a mediator between job stress and work–family conflict.

To test the moderated mediation model, after controlling for gender, years of teaching experience, and marriage, the self-efficacy moderation test was conducted using the SPSS PROCESS macro (see Table 3). As the table shows, the product (interaction term) of work–family conflict and resilience positively affected job burnout (*β* = 0.11, *t* = 2.03, *p* < 0.01), as did the product (interaction term) of work–family conflict and resilience onwork–family conflict (*β* = 0.23, *t* = 3.46, *p* < 0.01), thereby verifying H3. This result suggests that resilience moderated not only the direct effect of work stress on burnout but also the predictive effect of work stress on work–family conflict. Then, this study plotted the predicted relationship between work–family conflict and burnout separately for low and high levels of resilience (M ± 1 SD). As Figure 2 shows, simple slope tests showed that for IPE teachers with low resilience, job stress exerted a greater impact on burnout (βsimple = 0.39, *p* < 0.001.). However, for IPE teachers with high resilience, job stress significantly predicted burnout, but to a much weaker extent (βsimple = 0.27, *p* < 0.001). These results illustrate that as the resilience of IPE teachers rose, the predictive effect of job stress on burnout gradually decreased. The simple slope tests (Figure 3) demonstrated that job stress exerted a greater impact on work–family conflict among less resilient IPE teachers (βsimple = 0.41, *p* < 0.001.). However, for those with high levels of resilience, job stress significantly predicted work–family conflict but to a much weaker extent (βsimple = 0.22, *p* < 0.001). As the resilience of IPE teachers rose, the predictive effect of job stress on work–family conflict gradually increased.

**Table 3:** Testing the moderated mediation effect of job stress on burnout.

Predictors	*R* ^2^	*F*	*β*	*t*
Model (job burnout)	0.4	30.18^***^		
Job stress			0.28	5.26^***^
Resilience			−0.25	−4.92^***^
Job stress × Resilience			0.11	2.03^**^
Model (WFC)				
Job stress			0.34	6.19^***^
Resilience			−0.28	−4.65^***^
Job stress × Resilience			0.23	3.46^**^

**p < 0.01; ***p < 0.001; WFC, work–family conflict.

## Discussion

This study has examined the (in)diret links between the burnout levels of IPE teachers and job stress, work–family conflict and resilience. The study aligns with research that investigates human potential as an essential aspect of sustainable working conditions (Di Fabio, 2017a). It has confirmed the relationship between job stress and burnout among Chinese IPE teachers, and demonstrates that conservation of resources theory applies to the Chinese context. Moreover, the study’s investigation of work–family conflict as a mediator of job stress and burnout has enriched knowledge of the overall mechanism in the Chinese context and beyond. More importantly, the finding that the resilience of IPE teachers moderated the linkage between job stress and burnout suggests that the impact of job stress on work–family conflict and job burnout may be mitigated through by interventions that aim to build resilience, which has been shown to play a crucial preventive role in the mechanism of job burnout.

The results showed that job stress was significantly and positively related to burnout. The pandemic has required IPE teachers to manage multiple demands from stakeholders including schools, colleagues, student affairs bodies and society more broadly. It is thus unsurprising that teachers in one study reported high levels of stress when face-to-face classes were suspended due to the pandemic and a significant increase in their workload, which was associated with physical symptoms (Rubilar and Oros, 2021). Likewise, Latin American teachers reported an increase in stress-linked psychophysical signs during the pandemic (Casimiro Urcos et al., 2020). The first hypothesis was thus verified and consistent with previous research findings (Teles et al., 2020).

The study also found that work–family conflict mediated between job stress and burnout among IPE teachers. During the pandemic levels of job stress exacerbated work–family conflict, which in turn affected burnout among teachers, highlighting the crucial mediating role of family. Previous studies had demonstrated how work–family conflict can affect personal development (Kreiner et al., 2009); during the pandemic itself, teachers in one study reported higher levels of job stress as (1) work and household tasks overlapped, (2) work schedules lost their familiarity and stability, (3) insufficient time was available for tasks and (4) organizational demands increased (Rubilar and Oros, 2021). Lacking the time and energy to complete tasks, teachers experienced work–family conflict (Ji and Yue, 2020). Resource conservation theory holds that teachers confronted by work–family conflict will divert their resources into maintaining harmony and balance in their relationships with colleagues and family members (LePine et al., 2004). However, if teachers’ time, energy, or psychological resources are depleted without replenishment, they can devote fewer resources to their work and may suffer job burn-out (Zhao et al., 2022). Teachers take on important roles at home and at work that are important causes of burnout. The findings of this study are consistent with those of previous studies.

The moderation analysis demonstrated that resilience moderated the path from job stress to work–family conflict and job burnout, underlining its ability to mitigate the connection between the first and final points on this path. This study found that the impact of job stress on work–family conflict and burnout diminished as the resilience of IPE teachers increased which is consistent with previous studies. Individual psychological resources such as resilience impact burnout (Hobfoll and Freedy, 2017), with more resilient teachers exposed to lower risks of the condition, possibly due to their ability to marshal motivational and cognitive resources to overcome obstacles and resolve conflicts, as previous studies have shown (Ferradás et al., 2019). Even under unfavorable lockdown conditions, resilient teachers are motivated to work hard and achieve their professional goals, demonstrating the flexibility to adapt to events (Sabouripour and Roslan, 2015); this belief will greatly influence their motivation and professional self-efficacy, thereby reducing job burnout (Cheung et al., 2011). One study also found that the resilience of teacher groups buffered the effects of challenging professional circumstances for teachers, but not other professionals (Pretsch et al., 2012). The findings of this study thus supported our hypothesis that resilience would play an important moderating role between job stress and job burnout. The results confirm the importance of attending closely to the psychological health of IPE teachers and building their resilience at every opportunity.

The well-being of workers is crucial to thedevelopment of healthier organizations and societies (Di Fabio, 2017b). Our findings highlight that resilience is a crucial resource in building sustainable working conditions for teachers, owing to its associations with reduced work–family conflict and lower vulnerability to burnout. Resilience is not an innate attribute but can be acquired and developed over a lifespan (Howard and Johnson, 2004). Faced with the threat of an epidemic and the pressure to teach online, IPE teachers can crucially protect themselves by cultivating psychological resilience. Educational administrations should therefore design and apply interventions that promote the ability of teachers to resist and recover from adverse circumstances. Resilience training should be embedded in preservice IPE teacher education programs and provided to enable new IPE teachers manage their work, avoid burnout, fulfill their duties, and restore or renew their professional commitment. Resilience training is particularly important for experienced IPE teachers, who are more likely to encounter burnout (Chen, 2014). It can activate their personal values, help channel their energy and increase their enthusiasm for education. Moreover, all sectors of society should work together to create family, work and social support networks that nurture IPE teachers’ resilience.

### Limitations and future research

Several limitations need to be considered when assessing the contribution of this study. First, the cross-sectional study design limits the possibility of making causal inferences about the relationship between job stress and burnout. Future research may benefit from examining this relationship using longitudinal designs to obtain stronger empirical evidence of causality. A second limitation was the study’s lack of differentiation between individual factors such as gender and years of teaching experience—another shortcoming that future studies should address. Finally, although the study tested the moderating role of resilience on the linkages between job stress, work–family conflict and job burnout, additional work is required to investigate the complex mechanisms by which resilience shields teachers from these negative work-related issues. Such investigations will promote the sustainable professional development of IPE teachers.

## Conclusion

This study has contributed to the knowledge of the relationship between job stress and burnout among IPE teachers in China during the recent pandemic. Its implications for alleviating the professional stress of these teachers and reducing their susceptibility to job burnout are highly significant. The results demonstrate that work–family conflict mediates the relationship between job stress and burnout among IPE teachers. Resilience moderates this relationship as well as the linkage between job stress and work–family conflict and should be cultivated to alleviate the effects of work–family conflict and prevent burnout.

## Data availability statement

The raw data supporting the conclusions of this article will be made available by the authors, without undue reservation.

## Ethics statement

The studies involving human participants were reviewed and approved by the Ethics Committee of East China Normal University. The patients/participants provided their written informed consent to participate in this study.

## Author contributions

All authors listed have made a substantial, direct, and intellectual contribution to the work and approved it for publication. All authors contributed to the article and approved the submitted version.

## Funding

This study was supported by The National Social Science Fund of the Chinese government [no. 21CKS007].

## Conflict of interest

The author declares that the research was conducted in the absence of any commercial or financial relationships that could be construed as a potential conflict of interest.

## Publisher’s note

All claims expressed in this article are solely those of the authors and do not necessarily represent those of their affiliated organizations, or those of the publisher, the editors and the reviewers. Any product that may be evaluated in this article, or claim that may be made by its manufacturer, is not guaranteed or endorsed by the publisher.
